# Age-Dependent Modulations of Resting State Connectivity Following Motor Practice

**DOI:** 10.3389/fnagi.2018.00025

**Published:** 2018-02-06

**Authors:** Elena Solesio-Jofre, Iseult A. M. Beets, Daniel G. Woolley, Lisa Pauwels, Sima Chalavi, Dante Mantini, Stephan P. Swinnen

**Affiliations:** ^1^Movement Control and Neuroplasticity Research Group, Department of Movement Sciences, KU Leuven, Leuven, Belgium; ^2^Department of Biological and Health Psychology, Autonomous University of Madrid, Madrid, Spain; ^3^Department of Health Sciences and Technology, ETH Zurich, Zurich, Switzerland; ^4^Department of Experimental Psychology, University of Oxford, Oxford, United Kingdom; ^5^Leuven Research Institute for Neuroscience and Disease, KU Leuven, Leuven, Belgium

**Keywords:** aging, resting state functional connectivity, motor learning, motor network, bimanual coordination

## Abstract

Recent work in young adults has demonstrated that motor learning can modulate resting state functional connectivity. However, evidence for older adults is scarce. Here, we investigated whether learning a bimanual tracking task modulates resting state functional connectivity of both inter- and intra-hemispheric regions differentially in young and older individuals, and whether this has behavioral relevance. Both age groups learned a set of complex bimanual tracking task variants over a 2-week training period. Resting-state and task-related functional magnetic resonance imaging scans were collected before and after training. Our analyses revealed that both young and older adults reached considerable performance gains. Older adults even obtained larger training-induced improvements relative to baseline, but their overall performance levels were lower than in young adults. Short-term practice resulted in a modulation of resting state functional connectivity, leading to connectivity increases in young adults, but connectivity decreases in older adults. This pattern of age differences occurred for both inter- and intra-hemispheric connections related to the motor network. Additionally, long-term training-induced increases were observed in intra-hemispheric connectivity in the right hemisphere across both age groups. Overall, at the individual level, the long-term changes in inter-hemispheric connectivity correlated with training-induced motor improvement. Our findings confirm that short-term task practice shapes spontaneous brain activity differentially in young and older individuals. Importantly, the association between changes in resting state functional connectivity and improvements in motor performance at the individual level may be indicative of how training shapes the short-term functional reorganization of the resting state motor network for improvement of behavioral performance.

## Introduction

A large body of research has shown reduced abilities in motor skill performance and learning with age (Swinnen, 1998; Serrien et al., 2000; Bangert et al., 2010; Seidler et al., 2010; Solesio-Jofre et al., 2014; Pauwels et al., 2015; Serbruyns et al., 2015). As almost every motor skill used in daily life requires practice before being efficiently implemented, it is crucial to understand the neural mechanisms by which motor skills are learned and how they are modified with age. Behavioral research has shown that the motor learning process follows different stages. First, during an early acquisition stage considerable improvement in performance is achieved within a relatively short time, i.e., within a session. Second, in a later phase, performance becomes stable, with subtler training-induced improvement that involves consolidation processes. This is achieved over a longer period of time, i.e., between sessions spread over a period of several weeks (Ungerleider et al., 2002; Floyer-Lea and Matthews, 2005).

Converging evidence suggests that, although older adults are still able to acquire new motor skills, they may experience difficulties with the consolidation of acquired representations that occur in the later phase of learning (Voelcker-Rehage and Alberts, 2007; Brown et al., 2009; Wilson et al., 2012; Fogel et al., 2014; Pauwels et al., 2015; Mary et al., 2017). Neuroimaging research has shown a functional reorganization of different neural networks subtending motor performance in young individuals, involving neural plasticity mechanisms (Ma et al., 2010; Albouy et al., 2015). Among other regions, these networks include both inter- and intra-hemispheric connections between the supplementary motor area (SMA), the premotor cortex (PM), and the primary motor cortex (M1) (Donchin et al., 1998; Byblow et al., 2007; O’Shea et al., 2007; Talelli et al., 2008; Hinder et al., 2011). However, a reduced motor plasticity with progressing age may be responsible for the observed deficits in consolidation processes (Todd et al., 2010; Freitas et al., 2011; May and Zwaan, 2017).

Besides task training-induced functional activation changes, recent research has devoted increasing attention to resting state functional connectivity (rs-FC) as a reliable indicator of functional reorganization of brain networks supporting different mental processes (Biswal et al., 1995; Fox and Raichle, 2007; Greicius, 2008; van den Heuvel and Hulshoff Pol, 2010; Ma et al., 2011; van Dijk et al., 2017). Resting state functional magnetic resonance imaging (rs-fMRI) measures the large-scale covariance of low frequency spontaneous fluctuations in the blood oxygen level-dependent (BOLD) signal during rest. The strength of the correlation reflects the degree of functional connectivity between two or more brain regions.

Resting state functional magnetic resonance imaging studies investigating changes in functional connectivity following motor learning, have demonstrated that rs-FC can be modulated within and between training sessions in young individuals (Woolley et al., 2015). In one of the earliest studies, Albert et al. (2009) found that initial learning modulated both a fronto-parietal and a cerebellar resting state network. In a more confined motor network, Tung et al. (2013) showed that initial motor learning modulated functional connectivity between the right and left motor cortices, exhibiting increases in post-task compared to pre-task periods. Looking into a later phase of learning, increases in rs-FC in the superior parietal cortex (Daselaar et al., 2010) and in the postcentral and supramarginal gyrus (Ma et al., 2011) were observed. In short, these and other studies have shown that both short-term (Waites et al., 2005; Barnes et al., 2009; Stevens et al., 2010; Gregory et al., 2014; Sami et al., 2014; Mary et al., 2017) and long-term (Buchel et al., 1999; Voss et al., 2008; Tambini et al., 2010; Taubert et al., 2011; Yoo et al., 2013; Hardwick et al., 2015; Sampaio-Baptista et al., 2015; Woolley et al., 2015; Mehrkanoon et al., 2016; Amad et al., 2017; May and Zwaan, 2017) learning effects can modulate rs-FC in young individuals.

Importantly, rs-FC has also been shown to correlate with motor improvement in young adults (Ma et al., 2011; Vahdat et al., 2011; Wu et al., 2014; Zhang et al., 2014), indicating that functional network reorganization can, to some extent, predict behavioral changes (Tambini et al., 2010; Deco and Corbetta, 2011; Wu et al., 2014). However, results about motor training-induced modulation of resting state networks in older adults are very scarce, with only one study to date showing age-related rs-FC changes following motor sequence learning (Mary et al., 2017).

**FIGURE 1:**
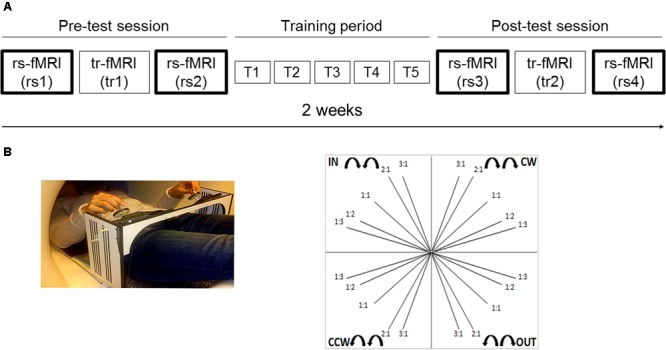
Experimental setup and task. **(A)** Schematic representation of the experimental setup. Two scan sessions occurred before (pre-test session) and after (post-test session) five training sessions (training period), distributed across 2 weeks. Each scan session included a rest scan (rs1 and rs3, respectively) before a task-related scan, a task-related scan (tr1 and tr2, respectively) and a rest scan after the task-related scan (rs2 and rs4, respectively). We mainly focused on rest scans (i.e., two runs within each scan session, four runs in total: rs1, rs2, rs3, and rs4); **(B)** The goal of the bimanual tracking task was to track a white target dot over a blue target line, presented on a screen, by rotating two dials with both hands simultaneously in one of four directional patterns: inward (IN), outward (OUT), clockwise (CW), and counter-clockwise manner (CCW); at five different relative frequency ratios: 1:1, 1:2, 1:3, 2:1, and 3:1 (left: right). This resulted in 20 different bimanual patterns and target line directions.

Here, we investigated whether and how motor skill acquisition and consolidation of a bimanual tracking task (BTT) (Solesio-Jofre et al., 2014; Serbruyns et al., 2015; Santos Monteiro et al., 2017) modulates rs-FC within a task-related motor network in young and older adults. Resting state activity was obtained across four scans (**Figure 1A**): two scans before a motor training protocol conducted over the course of 2 (one scan before a task-related fMRI scan and the other scan after the task-related fMRI scan), and two scans following completion of the motor training protocol (again, one scan before a task-related fMRI scan and the other scan after the task-related fMRI scan). The motor network was selected based on the results of a task-related fMRI study in which the same tracking task was used (Santos Monteiro et al., 2017). Due to the bimanual nature of the task, both inter- and intra-hemispheric functional connectivity were examined. It is well established that bimanual coordination relies on coupling between motor areas of both cerebral hemispheres (Serrien, 2008) through the corpus callosum (Gooijers et al., 2013; Gooijers and Swinnen, 2014). Moreover, learning a new bimanual coordination pattern results in changes in both intra- and inter-hemispheric coherence between pairs of motor regions, as shown by EEG studies (Andres et al., 1999; Gerloff and Andres, 2002; Serrien, 2009). In the current study, we specifically considered both homotopic (i.e., geometrically corresponding regions in each hemisphere) and non-homotopic inter-hemispheric, as well as right and left intra-hemispheric connectivity patterns. To the best of our knowledge, this is the first study examining motor training-induced modulations in both inter-and intra-hemispheric rs-FC as a function of aging during the early and late learning phase. Based on the study from Mary et al. (2017), we predicted an age-dependent reorganization of the motor network, not only immediately but also weeks after initial practice, with more prominent changes anticipated after the former.

Finally, to investigate the behavioral relevance of training-induced changes in functional connectivity, we correlated changes in rs-FC with bimanual task improvement over the course of learning. In accordance with previous studies (Ma et al., 2011; Taubert et al., 2011; Vahdat et al., 2011; Zhang et al., 2011, 2014; Wu et al., 2014), we expected both inter- and intra-hemispheric connectivity increases to be associated with improvements on the bimanual task.

## Materials and Methods

### Participants

Twenty-six healthy young adults (YA) and 25 older adults (OA) participated in the study. All participants had normal or corrected-to-normal vision, and were right-handed according to the Edinburgh Handedness Inventory (Oldfield, 1971). They were naive with respect to the experimental paradigm. None of the participants reported a history of neurological, psychiatric, or vascular disease. Older participants above 60 years old (*N* = 25) were screened for cognitive impairments with the Montreal Cognitive Assessment test (MoCA) using the standard cutoff score of 26 (Nasreddine et al., 2005). All participants obtained a score within normal limits (≥26, mean = 28.02, *SD* = 1.18, range = 26–30). Three YA were excluded from the analysis due to technical problems with the scanner. Four OA were excluded due to either brain atrophy/lesions, or inability to comply with task instructions. As a result, our final sample included 23 YA (age range = 17–26 years, mean age = 21.19, *SD* = 1.99, 12 females) and 21 OA (age range = 61–81 years, mean age = 68.85, *SD* = 5.89, 12 females). Informed consent was obtained before testing and participants were financially compensated for participation. The experiment was approved by the local ethics committee for biomedical research of KU Leuven (Belgium), and was performed in accordance with the Declaration of Helsinki (1964).

### Experimental Setup

Magnetic resonance imaging (MRI) occurred twice: before (pre-test session) and after (post-test session) five training sessions (training period), distributed across 2 weeks (see **Figure 1A**). Each training session had a total duration of 1 h. The rest-task-rest fMRI design of both scan sessions was identical with a total duration of 1.5 h. Therefore, the overall experimental procedure was as follows: the pre-test session included a rest scan (rs1), followed by a task-related scan (tr1), after which another rest scan (rs2) was obtained. Hence, rs1 and rs2 referred to the within pre-test session rest scans corresponding to the early phase of learning. The pre-test session was followed by a bimanual task training period of 2 weeks (T1, T2, T3, T4, T5). Following completion of 2 weeks of task training, the post-test session included a rest scan (rs3), followed by a task-related scan (tr2), and subsequently another rest scan (rs4). Hence, rs3 and rs4 referred to the within post-test session rest scans corresponding to the late phase of learning. The present study mainly focused on rest scans (i.e., two runs within each scan session, four runs in total: rs1, rs2, rs3, and rs4). All rest scans had the same protocol and lasted 8 min, in which participants were instructed to keep their eyes open and to fixate a target point. Results regarding the task-related fMRI study are published elsewhere (Santos Monteiro et al., 2017).

### Bimanual Tracking Task (BTT)

The BTT task was performed during the task-related scans. It enables the evaluation of bimanual coordination accuracy, relying on the execution of complex bimanual patterns (Sisti et al., 2011). This task requires intensive practice to successfully integrate the two separate limb movements into one common spatiotemporal pattern. Learning such a task involves breaking away from the natural tendency to move both limbs in phase with the same velocity, i.e., a 1:1 frequency ratio (Swinnen et al., 1997; Swinnen, 2002).

The goal of the BTT was to track a white target dot over a blue target line, presented on a screen, by rotating two dials with both hands simultaneously in one of four directional patterns: both hands rotated inward (IN) or outward (OUT) together, or in a clockwise (CW) or counter-clockwise manner (CCW) (Sisti et al., 2011, 2012; Gooijers et al., 2013). The left (L) and right (R) hands controlled movements on the ordinate and abscissa, respectively. To increase complexity of the task, each directional combination was performed at five different relative frequency ratios: 1:1, 1:2, 1:3, 2:1, and 3:1 (L:R). For example, a 1:2 ratio indicated that the left hand was required to rotate twice as slow as the right hand. This resulted in 20 different bimanual patterns and target line directions (**Figure 1B**).

Each trial started with the presentation of the single blue target line with a distinct orientation. At the origin of this line, in the center of the PC display, the white target dot was presented, after which it began to move along the blue target line, toward the peripheral endpoint. The target dot moved at a constant rate and for a total duration of 9 s. The beginning and end of the trajectory were marked with an auditory cue (126 ms, begin: 525 Hz, end: 442 Hz). The inter-trial interval was 3 s. The goal was to match the target trajectory as closely as possible.

Each BTT fMRI session consisted of 144 trials, divided equally across six runs, each of which lasted 6 min, with an inter-run interval of approximately 3 min. A run consisted of 24 target lines, presented in a pseudorandom order. The required frequency ratio was randomly distributed such that one third of trials required a 1:1 ratio, one-third required a 1:2 or 2:1 ratio and one-third required a 1:3 or 3:1 ratio. There were 96 “move” trials in which bimanual tracking was actively performed. The remaining trials were “no move” trials, containing the same information as the “move” trials but required no movement. They provided the baseline measure for the BOLD contrasts conducted in the task-related fMRI analysis (Santos Monteiro et al., 2017). Prior to the first MRI session, participants practiced the task briefly in a dummy scanner until the task was fully understood (∼10 min).

### Training Sessions

In the training sessions, participants were seated in front of a PC-screen (distance approximately 0.5 m) and performed the BTT. For each of the five training days, 10 blocks of 20 randomized trials corresponding to 20 bimanual patterns, i.e., five different frequency ratios in four directions, were performed.

### Kinematic Analyses

Data were recorded and analyzed with the Labview software (version 8.5, National Instruments, Austin, TX, United States). Offline analysis was carried out using Matlab R2011b. The *x* and *y* positions of the target dot and the cursor were sampled at 100 Hz. For each trial, we calculated the target deviation as a measure of accuracy, using the following multistep procedure: (a) Every 10 ms, the difference between the target position and the cursor position, *d*, was calculated, using the Euclidean distance:

$$\begin{array}{c} d=\sqrt{{\left(x_{2}\;-\;x_{1}\right)}^{2}\;+\;{\left(y_{2}\;-\;y_{1}\right)}^{2}} \end{array}$$

Where *x*_2_ and *y*_2_ refer to the position of the participant’s cursor on the *x*- and *y*-axis, respectively, and *x*_1_ and *y*_1_ correspond to the position of the target dot on the *x*- and *y*-axis, respectively. (b) At the end of each trial, the average of these distances was computed and defined as the trial’s target deviation, expressed in units (U). A target deviation equal to 0 U would indicate that during the whole trial, the cursor was precisely on top of the white target dot, representing perfect performance. Accordingly, greater target deviation scores reflect greater error and, hence, poorer performance.

To determine whether participants generally met the task requirements, all data were transformed into *z*-scores [(X-MEAN/SD)]. Trials were discarded from the analysis when *z* > 3 (outlier) and/or when only one hand moved (2.7 and 1.1% of all trials during BTT fMRI 1 and BTT fMRI 2, respectively). For each participant, the average error scores were computed for both scan sessions with and without augmented visual feedback, and these error scores were used as an indicator of bimanual performance accuracy.

### Statistical Analyses

Statistical analyses were performed using SPSS Version 22.0 (Armonk, NY, United States).

In accordance with previous results from our own group using the BTT task (Gooijers et al., 2013, 2016; Solesio-Jofre et al., 2014; Beets et al., 2015), movement directions (IN, OUT, CW, and CCW) were fully counterbalanced in the design and of no interest for the present analyses. Additionally, we collapsed trials into two levels: trials with the same (ISO, 1:1) and trials with different (N-ISO, 1:2, 1:3, 2:1, 3:1 collapsed) cycling frequency ratios.

Behavioral data acquired during both the pre- and post-test session were subjected to a 2 × 2 × 2 (age × scan session × frequency ratio) repeated measures ANOVA. Here, age (young, older) was the between-subject factor, and scan session (pre-test session and post-test session) and frequency ratio (ISO, N-ISO) were the within-subject factors.

The level of significance was set at *p* < 0.05. Significant effects were further explored using *post hoc* paired *t*-tests using Bonferroni correction for multiple comparisons. The partial eta squared statistic ($\eta_{p}^{2}$) was calculated as the effect size measure for main and interaction effects in the repeated measures ANOVA. According to Cohen (1992), $\eta_{p}^{2}$ values of 0.01, 0.06 and 0.13 represent small, medium and large effects, respectively.

### MRI Data Acquisition

Data acquisition, pre-processing, and analyses followed the same steps for the four resting state runs (rs1, rs2, rs3, and rs4). A Siemens 3-T Magnetom Trio MRI scanner (Siemens, Erlangen, Germany) with a 12 channel head coil was used. For anatomical details, a 3D high-resolution T1-weighted image was obtained first (magnetization prepared rapid gradient echo, time repetition/time echo = 2300/2.98 ms, 1 mm × 1 mm × 1.1 mm voxels, field of view (FOV) = 240 × 256, 160 sagittal slices), lasting 8 min. Then a field map was acquired to address local distortions.

Functional resting state data were acquired with a descending gradient echo planar imaging (EPI) pulse sequence for T_2_^∗^ - weighted images (repetition time = 3,000 ms; echo time = 30 ms; flip angle = 90°; 50 oblique axial slices each 2.8 mm thick; inter-slice gap = 0.028 mm; in-plane resolution 2.5 mm × 2.5 mm; 80 × 80 matrix, 160 volumes).

### MRI Data Pre-processing

Standard preprocessing procedures were performed using SPM8 (Statistical Parametric Mapping software, SPM: Wellcome Department of Imaging Neuroscience, London, United Kingdom^1^), which is implemented in Matlab 7.7 (The Mathworks, Natick, MA, United States).

Functional images were slice-time corrected to the middle slice (reference slice = 25), spatially realigned to the first image in the time series, normalized to the standard EPI template in Montreal Neurological Institute (MNI) space, and resampled into 3 mm isotropic voxels (Friston et al., 1995). Spatial smoothing was not applied in order to avoid introducing artificial local spatial correlations (Salvador et al., 2005; Achard et al., 2006; Achard and Bullmore, 2007).

We took additional preprocessing steps to remove spurious sources of variance. We defined a small, bilateral region of interest in the ventricles, a region of interest in the deep white matter, and one covering the whole brain; we then calculated the average signals in these three regions, which are typically referred to as cerebrospinal fluid, white matter and global signals (Fox et al., 2005, 2009). Next, we performed a regression analysis on the fMRI time-courses, modeling the three aforementioned signals and the parameters obtained by rigid body head motion realignment (Fox et al., 2005, 2009), as well as their temporal derivatives, as regressors.

Recently, there has been considerable discussion over the impact of head motion on rs-FC connectivity analyses. In addition to regressing out the three-dimensional motion parameters and their first derivatives, we also included regressors to deweight scans with a framewise displacement greater than 0.5 mm. A separate regressor was included for each outlier scan, with a 1 at the outlier time point and a zero at all other time points. Framewise displacement was calculated as the sum of the absolute scan to scan difference of the six translational and rotational realignment parameters (Power et al., 2014). Only 0.9% of all scans exceeded this threshold, and there was no significant difference in mean framewise displacement between the four resting state scans [one-way ANOVA: *F*(3,172) = 1.90, *p* = 0.14)].

The BOLD time course in each voxel was then temporally band-pass filtered (0.01–0.08 Hz) to reduce low-frequency drift and high-frequency noise.

### Region Definition

Candidate ROIs were generated from task-related fMRI scans (Santos Monteiro et al., 2017), in which the main aim was to explore the effects of aging on brain plasticity associated with motor learning while subjects performed the BTT. The main BOLD contrast of interest was bimanual visuomotor task performance (movement) vs. baseline condition (no movement) in young and older adults. Young and old *z*-score maps from the task-based fMRI study were combined to find overlapping ROIs by means of a conjunction analysis [young (bimanual visuomotor task > baseline) ∩ old (bimanual visuomotor task > baseline)] (Nichols et al., 2005). The statistical threshold was set to *p* < 0.05, FWE corrected for multiple comparisons and a minimal cluster size of 20 voxels.

**FIGURE 2:**
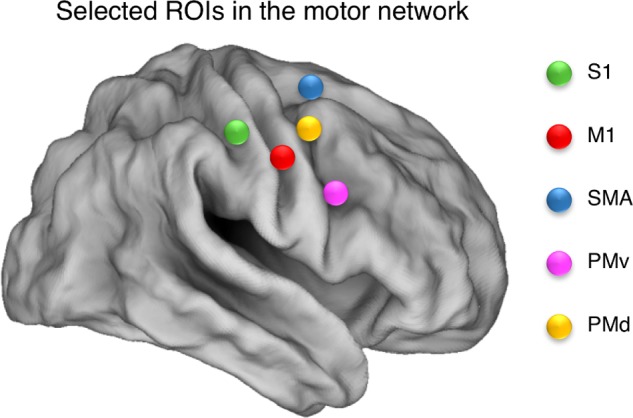
Selected ROIs in the motor network. Spherical ROIs were defined bilaterally for the following areas: SMA, supplementary motor area; PMd, dorsal premotor area; M1, primary motor cortex; S1, primary somatosensory area; PMv, ventral premotor area. The ROIs are illustrated over a cortical representation for the right hemisphere only.

To define regions for our resting state connectivity analysis, we chose the peak voxel with the highest *z*-score (*z* ≥ 4.10) in the positive group analysis. Our ROIs were composed of 6-mm radius spheres centered on these peak voxels and were created using the MarsBAR toolbox^2^. The size of the spheres was selected to ensure that they contained voxels that were significantly activated in all cases. We defined the following *a priori* ROIs: SMA (R, L); dorsal premotor area (PMd: R, L); ventral premotor area (PMv: R, L); primary motor cortex (M1: R, L); and primary somatosensory area (S1: R, L). ROI coordinates are listed in **Table 1**, and are illustrated in **Figure 2**.

**Table 1:** Regions defined for the resting state motor network.

Area	Hemisphere	*x*	*y*	*z*	*z*-score
**Movement > baseline**					
Supplementary motor area (SMA)	R	10	4	68	4.12
	L	–10	4	68	4.88
Dorsal premotor area (PMd)	R	28	–4	68	5.56
	L	–28	–4	68	6.63
Ventral premotor area (PMv)	R	54	8	34	6.06
	L	–54	8	34	4.31
Primary motor cortex (M1)	R	37	–21	58	8.02
	L	–37	–21	58	8.05
Primary somatosensory area (S1)	R	28	–40	52	4.64
	L	–28	–40	52	7.58

*Regions obtained after a conjunction analysis (young ∩ old) from a task-based fMRI study, *p*_*FWE*_ < 0.05, *z* ≥ 4.10. Six-mm radius spheres centered on *z*-peak voxels. R, right; L, left; SMA, supplementary motor area; PMd, dorsal premotor area; M1, primary motor cortex; S1, primary somatosensory area; PMv, ventral premotor area.*

### Functional Connectivity Analysis

For each subject and within each of the four resting state scans, regional mean time series were extracted by averaging the functional MRI time series across all voxels within each ROI. Then, the correlation strength between every pair of ROIs was calculated using Pearson correlation coefficients creating a functional network captured by a 10 × 10 correlation matrix. These Pearson correlation values were converted to *Z*-scores by Fischer’s *r*-to-*z* transformation (Zar, 1998), correcting the degrees of freedom for the autocorrelation in the time series (Shumway and Stoffer, 2006). Group-level correlation matrices were created by using a random-effects analysis across subjects (Ebisch et al., 2011; Pravata et al., 2011).

Next, we calculated the average connectivity score for a group of ROI pairs, which is the average of the component Fisher *Z*-scores for the corresponding ROI pairs. Specifically, we report four kinds of average functional connectivity (FC) scores, including homotopic inter-hemispheric FC, heterotopic inter-hemispheric FC, right intra-hemispheric FC and left intra-hemispheric FC. Average connectivity scores were subjected to repeated measures ANOVAs. We conducted a 2 × 2 × 2 × 2 (age × inter-hemispheric FC × scan session × scan location) repeated measures ANOVA, with age (young, older) as the between-subject factor and inter-hemispheric FC (homotopic, heterotopic), scan session (pre-test session, post-test session) and scan location (before task-related scan, after task-related scan) as the within-subject factors. Additionally, we conducted a 2 × 2 × 2 × 2 (age × intra-hemispheric FC × scan session × scan location) repeated measures ANOVA, with age (young, older) as the between-subject factor and intra-hemispheric FC (right, left), scan session (pre-test session, post-test session) and scan location (before task-related scan, after task-related scan) as the within-subject factors. All statistical tests were completed with alpha set at 0.05, and significant interaction effects were further explored by *post hoc* paired *t*-tests using Bonferroni correction for each repeated measures ANOVA conducted. The partial eta squared statistic ($\eta_{p}^{2}$) was calculated as the effect size measure for main and interaction effects in the repeated measures ANOVA and the size of the effects was interpreted according to Cohen (1992).

Finally, we calculated brain-behavior correlations to determine the extent to which training-induced changes in inter- and intra-hemispheric connectivity corresponded with the subsequent gain in behavioral performance, considering the entire sample of participants. We used two different calculations to quantify training-induced changes in inter- and intra-hemispheric connectivity during the early phase of learning and the overall learning process: (1) the difference in the average rs-FC between the second (rs2) and the first (rs1) resting state scans to study the effect of task learning during the early phase of learning; and (2) the difference in the average rs-FC between the last (rs4) and the first (rs1) resting state scans to investigate the effect of long-term practice.

Similarly, we used two different calculations to quantify the behavioral gain during the early phase of learning and the overall learning process: (a) the difference in the average target deviation between the last block of trials (15 trials in total) and the first block of trials (15 trials in total) in the first task-related fMRI scan (tr1) to study the effect of task practice during the early phase of learning; (b) the difference between the average target deviation in the last block of trials (15 in total) of the second task-related fMRI scan (tr2) and the first block of trials (15 in total) in tr1 to investigate the effect of long-term practice.

**FIGURE 3:**
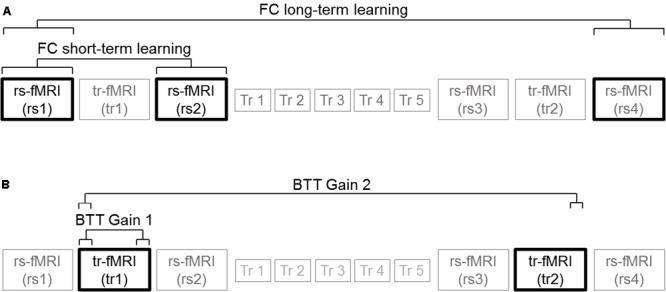
Brain-behavior correlations. **(A)** Functional connectivity measures: Connectivity changes extracted from rs2 minus rs1 (FC short-term learning), and also from rs4 minus rs1 (FC long-term learning) for inter- (homotopic, heterotopic) and intra-hemispheric (right, left) connectivity measures. **(B)** BTT gain measures: Last 15 trials of tr1 minus first 15 trials of tr1 (BTT Gain 1), and also last 15 trials of tr2 minus first 15 trials of tr1 (BTT Gain 2) for N-ISO conditions.

Hence, we performed brain-behavior correlations between the following functional connectivity measures: (A) Homotopic connections extracted from rs2 minus rs1 (Hm FC short-term learning) difference 1; (B) Heterotopic connections extracted from rs2 minus rs1 (Ht FC short-term learning); (C) Homotopic connections extracted from rs4 minus rs1 (Hm FC long-term learning); (D) Heterotopic connections extracted from rs4 minus rs1 (Ht FC long-term learning); (E) Right hemispheric connections extracted from rs2 minus rs1 (R FC short-term learning); (F) Left hemispheric connections extracted from rs2 minus rs1 (L FC short-term learning); (G) Right hemispheric connections extracted from rs4 minus rs1 (R FC long-term learning); (H) Left hemispheric connections extracted from rs4 minus rs1 (L FC long-term learning),

BTT gain measures were defined as follows:

(A) The last 15 trials of tr1 minus first 15 trials of tr1 for N-ISO condition (BTT Gain 1); (B) The last 15 trials of tr2 minus the first 15 trials of tr1 for N-ISO condition (BTT Gain 2). We focused on the N-ISO conditions as these represented new unfamiliar patterns requiring practice to improve proficiency whereas the ISO conditions reflected familiar patterns that constitute the default coordination modes (not requiring elaborate practice) (Sisti et al., 2011). Greater BTT gains reflect larger improvements in performance. **Figure 3** illustrates the correlations computed. Correlations surviving Bonferroni correction (*p* < 0.025) were considered significant.

## Results

### Kinematic Data

#### Scan Sessions

Motor performance during the scan sessions was assessed with a 2 × 2 × 2 (age × scan session × frequency ratio) repeated measures ANOVA for average target deviation. There was a main effect of age [*F*(1,42) = 49.59, *p* < 0.0001, $\eta_{p}^{2}$ = 0.54], with YA performing better than OA.

There was also a strong learning effect from pre- to post-training period, reflected by the main effect of scan session [*F*(1,42) = 109.8, *p* < 0.0001, $\eta_{p}^{2}$ = 0.72], indicating that performance improved as a result of training.

We also observed a main effect of frequency ratio [*F*(1,42) = 197.50, *p* < 0.0001, $\eta_{p}^{2}$ = 0.83], suggesting that subjects had more difficulty in performing the most difficult (N-ISO) as compared with the easiest (ISO) frequency ratios.

A significant age × scan session interaction effect [*F*(1,42) = 20.71, *p* < 0.001, $\eta_{p}^{2}$ = 0.33] indicated that, although both age groups were able to significantly improve their performance as a result of training [YA: *t*(22) = 8.95, *p* < 0.0001: OA: *t*(20) = 7.65, *p* < 0.0001], OA improved their performance to a higher degree as compared to YA from pre-test to post-test session. Furthermore, a significant age × frequency ratio interaction effect was observed [*F*(1,42) = 10.25, *p* = 0.003, $\eta_{p}^{2}$ = 0.20], reflecting that OA, but not YA, had more difficulty in performing the most difficult (N-ISO) relative to the easiest (ISO) condition [*t*(42) = -9.03, *p* < 0.0001].

#### Training Sessions

A 2 × 5 × 2 (age × training session × frequency ratio) repeated measures ANOVA was conducted for the average target deviation scores obtained across training days.

There was a main effect of age [*F*(1,42) = 33.94, *p* < 0.0001, $\eta_{p}^{2}$ = 0.48], indicating that the overall performance level of YA was better than the one of OA.

The main effect of training session was also significant [*F*(4,168) = 99.90, *p* < 0.0001, $\eta_{p}^{2}$ = 0.70], suggesting a strong practice effect. *Post hoc t*-tests revealed that the five sessions differed from each other (all *p* < 0.001). However, greater differences were observed for the first two sessions as compared to sessions 3, 4, and 5, suggesting that the practice effect was strongest at the first training sessions and a plateau effect was reached toward the final two sessions.

A significant main effect of frequency ratio [*F*(1,42) = 120.57, *p* < 0.0001, $\eta_{p}^{2}$ = 0.74] reflected greater error rates for N-ISO as compared to ISO ratio.

We observed a significant age × training session interaction effect [*F*(4,168) = 7.85, *p* < 0.0001, $\eta_{p}^{2}$ = 0.16]. *Post hoc t*-tests revealed that, although YA had a better performance than OA in all the five training sessions, these age differences were statistically greater during training session 1 compared to sessions 4 [*t*(42) = -3.29, *p* < 0.004] and 5 [*t*(42) = -3.76, *p* < 0.001], suggesting that as training progressed, the differences in performance between YA and OA decreased.

**FIGURE 4:**
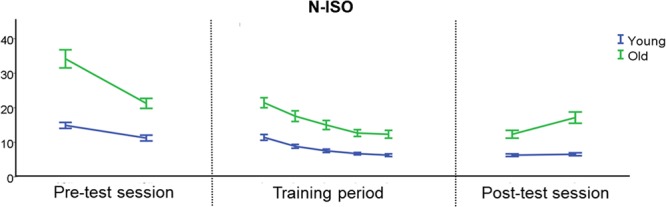
Behavioral performance during the scan and training sessions for the N-ISO condition. There was an initial reduction in target deviation error during the pre-test session, indicative of initial learning. During the training period, BTT performance became more stable, particularly during the last two training days. YA showed a more stable performance during the post-test session than OA, especially in the most difficult task condition (N-ISO). Error bars represent the standard error of the mean (SEM). N-ISO, non-isofrequency.

As not much learning was required for the ISO condition, **Figure 4** focuses on the behavioral performance during both the scan and training sessions for the N-ISO condition.

### Imaging Data

**FIGURE 5:**
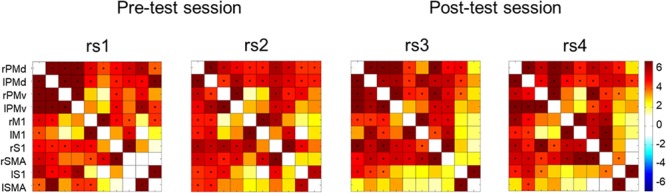
Correlation matrices across all participants showing the strength of functional connectivity between each pair of regions from the motor network for the four rest scans collected in the present study. Significant correlations (Bonferroni corrected probability, *p* < 0.001) are indicated with a black dot. Color bar on the right indicates *t*-values.

We studied low-frequency functional correlations associated with a task-specific motor network composed of 10 ROIs: left and right SMA, PMd, PMv, M1, and S1. We calculated Pearson correlation coefficients between each pair of ROIs across subjects, and within each of the four resting state scans. **Figure 5** shows the resulting 10 × 10 correlation matrix for each resting state scan, which reflects the strength of functional connectivity between each pair of regions. Next, we calculated average functional connectivity scores regarding four different groups of ROI pairs, including homotopic inter-hemispheric FC, heterotopic inter-hemispheric FC, right intra-hemispheric FC and left intra-hemispheric FC.

#### Modulations of Resting State Inter-Hemispheric Connectivity in Young and Older Adults throughout the Learning Process

**FIGURE 6:**
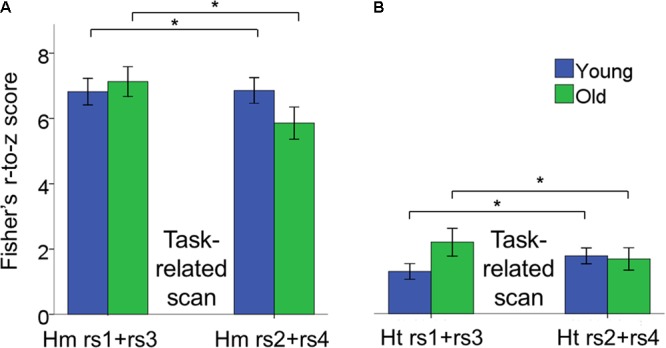
Bar plots showing the age × scan location interaction effect for inter-hemispheric functional connectivity. **(A)** Changes in connectivity in homotopic pairs of regions. **(B)** Changes in connectivity in heterotopic pairs of regions. In both cases, functional connectivity increased after task performance in YA, whereas it decreased in OA and we observed this pattern of results within both the pre- and post-test sessions. Moreover, homotopic functional connectivity was greater than heterotopic functional connectivity. Error bars represent SEM. Hm rs1+rs3, homotopic rest scans before task-related scans; Hm rs2+rs4, homotopic rest scans after task-related scans; Ht rs1+rs3, heterotopic rest scans before task-related scans; Ht rs2+rs4, heterotopic rest scans after task-related scans.

The 2 × 2 × 2 × 2 (age × inter-hemispheric FC × scan session × scan location) repeated measures ANOVAs revealed a main effect of inter-hemispheric functional connectivity [*F*(1,42) = 247.26, *p* < 0.0001, $\eta_{p}^{2}$ = 0.86], with greater homotopic than heterotopic connectivity values (Hm: 6.68 ± 0.29, Ht: 2.52 ± 0.18) (**Figure 6**). Main effects of age group, scan session and phase were not significant.

A significant age × scan location interaction indicated that young and older adults showed a different pattern of rs-FC change as a function of performance of the BTT both during the pre- and post-test sessions [*F*(1,42) = 9.01, *p* = 0.005, $\eta_{p}^{2}$ = 0.18]. Subsequent *post hoc* unpaired *t*-tests demonstrated that rs-FC increased after practicing the BTT, that is from rs1 to rs2 and from rs3 to rs4, in YA, whereas OA showed the opposite pattern [*t*(42) = -2.21, *p* = 0.002. YA, pre-task rs: 4.48 ± 0.41, post-task rs: 4.71 ± 0.28; OA, pre-task rs: 5.03 ± 0.33, post-task rs: 4.16 ± 0.30)]. This effect was true for both homotopic and heterotopic connections. **Figure 6** depicts the age × scan location interaction. None of the remaining interactions reached significance.

#### Modulations of Resting State Intra-Hemispheric Connectivity in Young and Older Adults throughout the Learning Process

The 2 × 2 × 2 × 2 (age × intra-hemispheric FC × scan session × scan location) repeated measures ANOVAs revealed no significant main effects of age, intra-hemispheric FC, scan session and learning phase.

**FIGURE 7:**
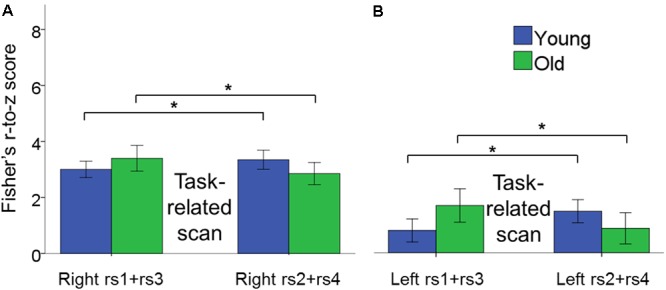
Bar plots show the age × scan location interaction effect for intra-hemispheric functional connectivity. **(A)** Changes in connectivity in right hemisphere pairs of regions. **(B)** Changes in connectivity in left hemisphere pairs of regions. In both cases, functional connectivity increased after task performance in YA, whereas it decreased after task performance in OA within pre- and post-test sessions. Error bars represent SEM.

There was a significant age × scan location interaction, in which young and older adults showed different patterns of rs-FC change as a function of task practice [*F*(1,42) = 6.21, *p* = 0.017, $\eta_{p}^{2}$ = 0.13]. Subsequent *post hoc* unpaired *t*-tests demonstrated that rs-FC increased after BTT performance in YA, that is from rs1 to rs2 and from rs3 to rs4, whereas OA exhibited the opposite pattern [*t*(42) = -2.56, *p* = 0.003. YA, pre-task rs: 2.75 ± 0.22, post-task rs: 3.15 ± 0.23; OA, pre-task rs: 3.23 ± 0.39, post-task rs: 2.70 ± 0.32)]. Of note, this is the same pattern as previously observed for inter-hemispheric functional connectivity. Moreover, this age-related difference in the pattern of functional connectivity occurred for both left and right hemisphere connections. **Figure 7** depicts the age × scan location interaction.

We also observed a significant intra-hemispheric functional connectivity × scan session interaction effect, indicating that functional connectivity within the right and left hemisphere showed a differential change from pre- to post-test session. [*F*(1,42) = 4.55, *p* = 0.04, $\eta_{p}^{2}$ = 0.10]. Subsequent *post hoc* paired *t*-tests revealed training-related increases in functional connectivity in the right hemisphere, but not in the left hemisphere, after the training period [*t*(43) = 2.62, *p* = 0.004; Right post-test session: 3.41 ± 0.32; Right pre-test session: 2.89 ± 0.24]. None of the remaining main and interaction effects reached significance.

### Correlation between Resting State Functional Connectivity and Behavior

We tested whether changes in rs-FC corresponded with gains in behavioral performance as a general tendency across both age groups.

**FIGURE 8:**
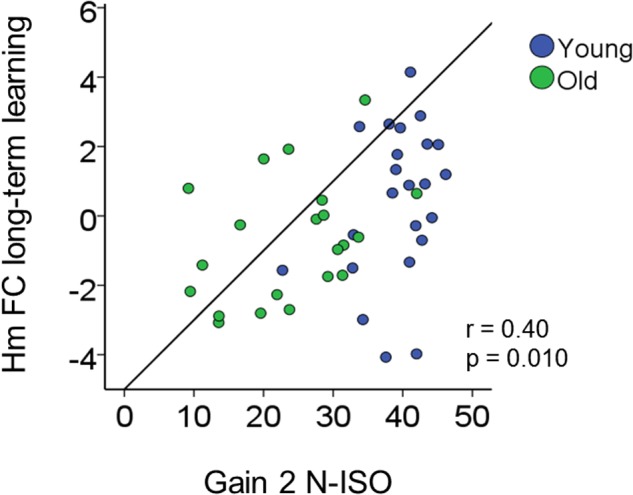
Brain connectivity-behavior correlation. Scatter plot representing the significant correlation surviving Bonferroni correction (*p* < 0.025) between rs-FC change (*y*-axis) and bimanual coordination gain (*x*-axis). *r*, Pearson coefficient.

Increases in inter-hemispheric connectivity for both homotopic (Hm FC long-term learning) and heterotopic (Ht FC long-term learning) connections correlated with greater gains in BTT performance (BTT Gain 2 N-ISO) (Hm: *r* = 0.40, *p* = 0.010; Ht: *r* = 0.30, *p* = 0.04). Of note, the first result survived Bonferroni correction (*p* < 0.013), whereas the second did not. None of the remaining correlations reached significance. See **Table 2** and **Figure 8** for further details.

**Table 2:** Correlations between inter- and intra-hemispheric rs-FC changes and BTT gains.

	Gain 1 N-ISO	Gain 2 N-ISO
Hm FC short-term learning	*r* = 0.23	*r* = 0.19
	*p* = 0.13	*p* = 0.21
Ht FC short-term learning	*r* = 0.02	*r* = 0.15
	*p* = 0.90	*p* = 0.34
Hm FC long-term learning	*r* = 0.21	***r* = 0.40**
	*p* = 0.16	***p* = 0.01**
Ht FC long-term learning	*r* = -0.06	***r* = 0.30**
	*p* = 0.68	*p* = 0.04
R FC short-term learning	*r* = 0.03	*r* = 0.12
	*p* = 0.85	*p* = 0.44
L FC short-term learning	*r* = 0.12	*r* = 0.19
	*p* = 0.45	*p* = 0.22
R FC long-term learning	*r* = 0.04	*r* = 0.26
	*p* = 0.81	*p* = 0.09
L FC long-term learning	*r* = -0.07	*r* = 0.18
	*p* = 0.66	*p* = 0.25

*Increases in inter-hemispheric connectivity from the first to the last resting state scan for both homotopic and heterotopic connections (Hm FC long-term learning, Ht FC long-term learning) correlated significantly with greater gains in BTT performance from the first 15 trials of the first task-related scan to the last 15 trials of the second task-related scan in the N-ISO condition (Gain 2). Only the bolded values corresponding to the correlation between homotopic rs-FC change and Gain 2 survived Bonferroni correction (*p* < 0.025). r, Pearson coefficient; *p*, probability value.*

## Discussion

We investigated whether learning a bimanual tracking task modulates rs-FC in the early and late phase of practice and whether this has behavioral relevance. Compared with YA, OA showed a lower motor performance in general, but a larger improvement relative to baseline after 2 weeks of training. Short-term practice effects achieved within pre- and post-test sessions modulated rs-FC, leading to connectivity increases in YA, but connectivity decreases in OA. This pattern of age differences occurred for both inter- and intra-hemispheric connections related to the motor network. We did not observe age-related modulations of long-term practice (i.e., from pre- to post-test session) on interhemispheric rs-FC at the group level. However, long-term training-induced increases were observed in intra-hemispheric connectivity in the right hemisphere (across both age groups). Finally, changes in inter-hemispheric functional connectivity from the start to end of practice correlated with training-induced motor improvement, underscoring the behavioral significance of rs-fMRI for prediction of motor skill learning. We discuss these findings in detail below.

### Aging Effects on Bimanual Coordination Learning

Kinematic analyses revealed that older individuals encountered particular difficulties with performing the bimanual tasks with different frequency ratios. This is a consequence of the higher complexity of these tasks. Strikingly, we observed greater performance improvement in older compared to young adults, which is in accordance with previous studies (Wishart et al., 2002; Voelcker-Rehage and Willimczik, 2006). This may be a consequence of the lower performance levels of older adults at baseline, giving rise to a larger window for improvement. However, both age groups showed a similar overall pattern of improvement: a stronger practice effect during the initial phase of learning (pre-test scan session), followed by a plateau toward the final phase (post-test scan session), reflecting relatively stable performance.

### Functional Connectivity Changes in Young and Older Adults as a Result of Short-Term Practice

We consistently observed an age-related change in rs-FC after short-term practice both during the early and later stages of learning (pre- and post-test sessions, respectively). Specifically, we observed increases in functional connectivity in young adults but decreases in older adults after task performance. Hence, task practice induced a differential short-term functional reorganization within the resting brain in young and older individuals. However, we did not observe an age-related functional reorganization of our motor network over the longer course of training across 2 weeks (from pre- to post-test session). This implies that the involvement in the training protocol did not induce long-term changes in rs-FC, at least not significantly different between both age groups. Hence, our pattern of results does not support our initial hypothesis of an age-dependent reorganization of the motor network that is more pronounced during the early (pre-test session) compared to the later stage of learning (post-test session), with the most dramatic changes in motor performance occurring in the former stage (Mary et al., 2017).

The findings observed in young adults are generally in accordance with previous research showing short-term effects on rs-FC after task practice (Peltier et al., 2005; Waites et al., 2005; Boonstra et al., 2007; Houweling et al., 2008; Barnes et al., 2009; Stevens et al., 2010; Klingner et al., 2012; Tung et al., 2013). In this regard, it seems reasonable to suggest that the short-term rs-FC increases following the execution of the motor task observed in young adults may reflect tighter communication between motor network areas. Although speculative, these processes may entail sensorimotor integration (Loayza et al., 2011; Ma et al., 2011; Wu et al., 2014) and short-term storage of visuomotor skills (Johnson-Frey et al., 2005; Ma et al., 2011).

The rs-FC decreases due to short-term practice, as observed in older adults, are more difficult to understand. In an attempt to come to grips with this finding, it is important to bear in mind that network organization differs between young and older adults and this pertains to within-network as well as between-network rs-FC. Previous work has shown that rs-FC within the motor network is higher in older as compared to younger adults and this is negatively associated with motor performance (Solesio-Jofre et al., 2014). Furthermore, FC among the different resting state networks is also increased in older adults, pointing to reduced overall network segregation (Geerligs et al., 2014; Chan et al., 2017; King et al., 2017), associated with poorer cognitive performance or no performance benefits at all (Nashiro et al., 2017). This is supportive of a dedifferentiation process, referring to an age-related diminished specificity in the cortical response to some stimulus categories and a reduction in the fidelity of neural representations (Grady et al., 1992; Park et al., 2004; Voss et al., 2008). Against the backdrop of these age-related changes, it is conceivable that training led to a reorganization of the motor network by strengthening interactions with other networks that have functional relevance for the task (such as attention or executive networks) and reducing interactions with other networks that are less or not relevant for task performance (such as the default mode network). This process of increasing efficiency of brain activity may be associated with a temporary reduction in FC between areas of the motor network, as observed in our findings. However, this is a hypothetical account that requires confirmation in future research.

### Age-Related Modulations of Inter- and Intra-Hemispheric Functional Connectivity

Interestingly, we observed the same pattern of age group differences for both inter- and intra-hemispheric connections. Regarding the former, it is important to remark that we observed greater homotopic relative to heterotopic functional connectivity values. Increased functional and structural homotopic connections have been commonly reported during the acquisition of bimanual coordination skills. On the basis of other studies employing rs-fMRI, this tendency for homotopic regions to exhibit stronger functional connectivity relative to heterotopic regions is more prevalent for regions of the adult human brain, such as motor, somatosensory, visual and subcortical regions (Zuo et al., 2010; Ruddy et al., 2017). In the same vein, previous research in non-human primates (Rouiller et al., 1994; Dancause et al., 2007) and humans (Ruddy et al., 2017) has reported that the largest proportions of interhemispheric fibers connecting to M1, SMA and PM originate in their homologous region in the contralateral hemisphere.

Finally, we observed that the strength of rs-FC within the right hemisphere increased after 2 weeks of training in both age groups, highlighting the importance of increased interregional interactions in the right hemisphere during motor skill learning in general and bimanual skill learning in particular (Halsband and Lange, 2006; Ma et al., 2011). This finding may reflect increased functional interactions among the right hemisphere motor areas to control the less skilled non-dominant hand as part of the unified bimanual kinematic chain. Successful coordination is contingent on the cooperation between both hemispheres/hands and this requires elevation of non-dominant hand function to better interact with the dominant hand.

### Behavioral Relevance for Learning-Related Modulations in Functional Connectivity

Even though neither of the groups exhibited a change in interhemispheric rs-FC across long-term practice, we observed an association between FC change and behavioral change across long-term practice at the individual level. Increases in the strength of inter-hemispheric connections across the 2-week period were associated with higher motor improvement in both young and older individuals, also across the 2-week period, suggesting that the resting state motor network may support the functional reorganization of the motor system in order to improve behavioral performance and ultimately, motor consolidation. This is in accordance with previous studies suggesting a role for resting state networks in terms of keeping active relevant functional systems to improve behavioral performance (Raichle et al., 2001; Miall and Robertson, 2006; van den Heuvel and Hulshoff Pol, 2010). Specifically, resting state networks become relevant functional units that may be recruited whenever a task needs extra support to be successfully performed.

In the light of these results, we suggest that resting state networks do not simply reflect physiological markers of anatomical pathways, but represent highly efficient modules of brain organization, somehow capable of predicting behavioral performance and improvement following motor learning.

## Conclusion

In this study, we provided behavioral evidence that motor learning capacity is preserved in aging. Furthermore, we demonstrated that shorter-term practice modulates the resting state differentially in young and older individuals. On the one hand, increased within-network connectivity after task practice observed in young adults may be indicative of enhanced interactions between motor network areas that are engaged in motor learning. On the other hand, an age-related reduction in within-network connectivity after task practice may be an indirect consequence of how the motor network interacts with other networks to optimize overall brain activity for task performance. Older adults may necessitate extra resources to learn the motor task, becoming more dependent on cognitive processes embodied in motor learning. Hence, further research is warranted to shed light on the behavioral relevance of functional interactions across the motor, memory and attentional networks during the resting state.

## Ethics Statement

This protocol was approved by the local ethics committee for biomedical research and subjects gave written informed consent prior to participation.

## Author Contributions

ES-J performed the data analyses and wrote the manuscript. IB conducted the data collection. DW helped with data analyses and language check. LP helped with behavioral analysis. SC helped with behavioral analysis. DM developed the pipeline for data analysis and helped with the writing of the manuscript. SS is the principal investigator of this project and helped with the writing of the manuscript.

## Conflict of Interest Statement

The authors declare that the research was conducted in the absence of any commercial or financial relationships that could be construed as a potential conflict of interest.

## Footnotes

1 http://www.fil.ion.ucl.ac.uk/spm/

2 http://marsbar.sourceforge.net

## References

[B1] Achard S. Bullmore E. (2007). Efficiency and cost of economical brain functional networks. *PLOS Comput. Biol.* 3:e17. 10.1371/journal.pcbi.0030017 17274684 PMC1794324

[B2] Achard S. Salvador R. Whitcher B. Suckling J. Bullmore E. (2006). A resilient, low-frequency, small-world human brain functional network with highly connected association cortical hubs. *J. Neurosci.* 26 63–72. 10.1523/JNEUROSCI.3874-05.2006 16399673 PMC6674299

[B3] Albert N. B. Robertson E. M. Miall R. C. (2009). The resting human brain and motor learning. *Curr. Biol.* 19 1023–1027. 10.1016/j.cub.2009.04.028 19427210 PMC2701987

[B4] Albouy G. Fogel S. King B. R. Laventure S. Benali H. Karni A. (2015). Maintaining vs. enhancing motor sequence memories: respective roles of striatal and hippocampal systems. *Neuroimage* 108 423–434. 10.1016/j.neuroimage.2014.12.049 25542533

[B5] Amad A. Seidman J. Draper S. B. Bruchhage M. M. K. Lowry R. G. Wheeler J. (2017). Motor learning induces plasticity in the resting brain-drumming up a connection. *Cereb. Cortex* 27 2010–2021. 10.1093/cercor/bhw048 26941381

[B6] Andres F. G. Mima T. Schulman A. E. Dichgans J. Hallett M. Gerloff C. (1999). Functional coupling of human cortical sensorimotor areas during bimanual skill acquisition. *Brain* 122 855–870. 10.1093/brain/122.5.855 10355671

[B7] Bangert A. S. Reuter-Lorenz P. A. Walsh C. M. Schachter A. B. Seidler R. D. (2010). Bimanual coordination and aging: neurobehavioral implications. *Neuropsychologia* 48 1165–1170. 10.1016/j.neuropsychologia.2009.11.013 19941878 PMC2828502

[B8] Barnes A. Bullmore E. T. Suckling J. (2009). Endogenous human brain dynamics recover slowly following cognitive effort. *PLOS ONE* 4:e6626. 10.1371/journal.pone.0006626 19680553 PMC2721686

[B9] Beets I. A. Gooijers J. Boisgontier M. P. Pauwels L. Coxon J. P. Wittenberg G. (2015). Reduced neural differentiation between feedback conditions after bimanual coordination training with and without augmented visual feedback. *Cereb. Cortex* 25 1958–1969. 10.1093/cercor/bhu005 24501382

[B10] Biswal B. Yetkin F. Z. Haughton V. M. Hyde J. S. (1995). Functional connectivity in the motor cortex of resting human brain using echo-planar MRI. *Magn. Reson. Med.* 34 537–541. 10.1002/mrm.1910340409 8524021

[B11] Boonstra T. W. Daffertshofer A. Breakspear M. Beek P. J. (2007). Multivariate time-frequency analysis of electromagnetic brain activity during bimanual motor learning. *Neuroimage* 36 370–377. 10.1016/j.neuroimage.2007.03.012 17462913

[B12] Brown L. E. Wilson E. T. Gribble P. L. (2009). Repetitive transcranial magnetic stimulation to the primary motor cortex interferes with motor learning by observing. *J. Cogn. Neurosci.* 21 1013–1022. 10.1162/jocn.2009.21079 18702578

[B13] Buchel C. Coull J. T. Friston K. J. (1999). The predictive value of changes in effective connectivity for human learning. *Science* 283 1538–1541. 10.1126/science.283.5407.1538 10066177

[B14] Byblow W. D. Coxon J. P. Stinear C. M. Fleming M. K. Williams G. Muller J. F. (2007). Functional connectivity between secondary and primary motor areas underlying hand-foot coordination. *J. Neurophysiol.* 98 414–422. 10.1152/jn.00325.2007 17507503

[B15] Chan M. Y. Alhazmi F. H. Park D. C. Savalia N. K. Wig G. S. (2017). Resting-state network topology differentiates task signals across the adult life span. *J. Neurosci.* 37 2734–2745. 10.1523/JNEUROSCI.2406-16.2017 28174333 PMC5354325

[B16] Cohen J. (1992). A power primer. *Psychol. Bull.* 112 155–159. 10.1037/0033-2909.112.1.155 19565683

[B17] Dancause N. Barbay S. Frost S. B. Mahnken J. D. Nudo R. J. (2007). Interhemispheric connections of the ventral premotor cortex in a new world primate. *J. Comp. Neurol.* 505 701–715. 10.1002/cne.21531 17948893 PMC3266721

[B18] Daselaar S. M. Huijbers W. de Jonge M. Goltstein P. M. Pennartz C. M. (2010). Experience-dependent alterations in conscious resting state activity following perceptuomotor learning. *Neurobiol. Learn. Mem.* 93 422–427. 10.1016/j.nlm.2009.12.009 20045076

[B19] Deco G. Corbetta M. (2011). The dynamical balance of the brain at rest. *Neuroscientist* 17 107–123. 10.1177/1073858409354384 21196530 PMC4139497

[B20] Donchin O. Gribova A. Steinberg O. Bergman H. Vaadia E. (1998). Primary motor cortex is involved in bimanual coordination. *Nature* 395 274–278. 10.1038/26220 9751054

[B21] Ebisch S. J. Gallese V. Willems R. M. Mantini D. Groen W. B. Romani G. L. (2011). Altered intrinsic functional connectivity of anterior and posterior insula regions in high-functioning participants with autism spectrum disorder. *Hum. Brain Mapp.* 32 1013–1028. 10.1002/hbm.21085 20645311 PMC6870194

[B22] Floyer-Lea A. Matthews P. M. (2005). Distinguishable brain activation networks for short- and long-term motor skill learning. *J. Neurophysiol.* 94 512–518. 10.1152/jn.00717.2004 15716371

[B23] Fogel S. M. Albouy G. Vien C. Popovicci R. King B. R. Hoge R. (2014). fMRI and sleep correlates of the age-related impairment in motor memory consolidation. *Hum. Brain Mapp.* 35 3625–3645. 10.1002/hbm.22426 24302373 PMC6869653

[B24] Fox M. D. Raichle M. E. (2007). Spontaneous fluctuations in brain activity observed with functional magnetic resonance imaging. *Nat. Rev. Neurosci.* 8 700–711. 10.1038/nrn2201 17704812

[B25] Fox M. D. Snyder A. Z. Vincent J. L. Corbetta M. Van Essen D. C. Raichle M. E. (2005). The human brain is intrinsically organized into dynamic, anticorrelated functional networks. *Proc. Natl. Acad. Sci. U.S.A.* 102 9673–9678. 10.1073/pnas.0504136102 15976020 PMC1157105

[B26] Fox M. D. Zhang D. Snyder A. Z. Raichle M. E. (2009). The global signal and observed anticorrelated resting state brain networks. *J. Neurophysiol.* 101 3270–3283. 10.1152/jn.90777.2008 19339462 PMC2694109

[B27] Freitas C. Perez J. Knobel M. Tormos J. M. Oberman L. Eldaief M. (2011). Changes in cortical plasticity across the lifespan. *Front. Aging Neurosci.* 3:5 10.3389/fnagi.2011.00005 PMC3079175 21519394

[B28] Friston K. J. Frith C. D. Frackowiak R. S. Turner R. (1995). Characterizing dynamic brain responses with fMRI: a multivariate approach. *Neuroimage* 2 166–172. 10.1006/nimg.1995.1019 9343599

[B29] Geerligs L. Maurits N. M. Renken R. J. Lorist M. M. (2014). Reduced specificity of functional connectivity in the aging brain during task performance. *Hum. Brain Mapp.* 35 319–330. 10.1002/hbm.22175 22915491 PMC6869200

[B30] Gerloff C. Andres F. G. (2002). Bimanual coordination and interhemispheric interaction. *Acta Psychol.* 110 161–186. 10.1016/S0001-6918(02)00032-X 12102104

[B31] Gooijers J. Beets I. A. Albouy G. Beeckmans K. Michiels K. Sunaert S. (2016). Movement preparation and execution: differential functional activation patterns after traumatic brain injury. *Brain* 139(Pt 9) 2469–2485. 10.1093/brain/aww177 27435093

[B32] Gooijers J. Caeyenberghs K. Sisti H. M. Geurts M. Heitger M. H. Leemans A. (2013). Diffusion tensor imaging metrics of the corpus callosum in relation to bimanual coordination: effect of task complexity and sensory feedback. *Hum. Brain Mapp.* 34 241–252. 10.1002/hbm.21429 22021056 PMC6869984

[B33] Gooijers J. Swinnen S. P. (2014). Interactions between brain structure and behavior: the corpus callosum and bimanual coordination. *Neurosci. Biobehav. Rev.* 43 1–19. 10.1016/j.neubiorev.2014.03.008 24661987

[B34] Grady C. L. Haxby J. V. Horwitz B. Schapiro M. B. Rapoport S. I. Ungerleider L. G. (1992). Dissociation of object and spatial vision in human extrastriate cortex: age-related changes in activation of regional cerebral blood flow measured with [(15) o]water and positron emission tomography. *J. Cogn. Neurosci.* 4 23–34. 10.1162/jocn.1992.4.1.23 23967855

[B35] Gregory M. D. Agam Y. Selvadurai C. Nagy A. Vangel M. Tucker M. (2014). Resting state connectivity immediately following learning correlates with subsequent sleep-dependent enhancement of motor task performance. *Neuroimage* 102(Pt 2) 666–673. 10.1016/j.neuroimage.2014.08.044 25173415 PMC4252600

[B36] Greicius M. (2008). Resting-state functional connectivity in neuropsychiatric disorders. *Curr. Opin. Neurol.* 21 424–430. 10.1097/WCO.0b013e328306f2c5 18607202

[B37] Halsband U. Lange R. K. (2006). Motor learning in man: a review of functional and clinical studies. *J. Physiol. Paris* 99 414–424. 10.1016/j.jphysparis.2006.03.007 16730432

[B38] Hardwick R. M. Lesage E. Eickhoff C. R. Clos M. Fox P. Eickhoff S. B. (2015). Multimodal connectivity of motor learning-related dorsal premotor cortex. *Neuroimage* 123 114–128. 10.1016/j.neuroimage.2015.08.024 26282855 PMC4780681

[B39] Hinder M. R. Schmidt M. W. Garry M. I. Carroll T. J. Summers J. J. (2011). Absence of cross-limb transfer of performance gains following ballistic motor practice in older adults. *J. Appl. Physiol.* 110 166–175. 10.1152/japplphysiol.00958.2010 21088207

[B40] Houweling S. Daffertshofer A. van Dijk B. W. Beek P. J. (2008). Neural changes induced by learning a challenging perceptual-motor task. *Neuroimage* 41 1395–1407. 10.1016/j.neuroimage.2008.03.023 18485745

[B41] Johnson-Frey S. H. Newman-Norlund R. Grafton S. T. (2005). A distributed left hemisphere network active during planning of everyday tool use skills. *Cereb. Cortex* 15 681–695. 10.1093/cercor/bhh169 15342430 PMC1364509

[B42] King B. R. van Ruitenbeek P. Leunissen I. Cuypers K. Heise K. F. Santos Monteiro T. (2017). Age-related declines in motor performance are associated with decreased segregation of large-scale resting state brain networks. *Cereb. Cortex* 9 1–13. 10.1093/cercor/bhx297 29136114 PMC6215458

[B43] Klingner C. M. Volk G. F. Brodoehl S. Burmeister H. P. Witte O. W. Guntinas-Lichius O. (2012). Time course of cortical plasticity after facial nerve palsy: a single-case study. *Neurorehabil. Neural Repair* 26 197–203. 10.1177/1545968311418674 21875890

[B44] Loayza F. R. Fernandez-Seara M. A. Aznarez-Sanado M. Pastor M. A. (2011). Right parietal dominance in spatial egocentric discrimination. *Neuroimage* 55 635–643. 10.1016/j.neuroimage.2010.12.011 21147233

[B45] Ma L. Narayana S. Robin D. A. Fox P. T. Xiong J. (2011). Changes occur in resting state network of motor system during 4 weeks of motor skill learning. *Neuroimage* 58 226–233. 10.1016/j.neuroimage.2011.06.014 21689765 PMC3144281

[B46] Ma L. Wang B. Narayana S. Hazeltine E. Chen X. Robin D. A. (2010). Changes in regional activity are accompanied with changes in inter-regional connectivity during 4 weeks motor learning. *Brain Res.* 1318 64–76. 10.1016/j.brainres.2009.12.073 20051230 PMC2826520

[B47] Mary A. Wens V. Op de Beeck M. Leproult R. De Tiege X. Peigneux P. (2017). Age-related differences in practice-dependent resting-state functional connectivity related to motor sequence learning. *Hum. Brain Mapp.* 38 923–937. 10.1002/hbm.23428 27726263 PMC6866841

[B48] May C. M. Zwaan B. J. (2017). Relating past and present diet to phenotypic and transcriptomic variation in the fruit fly. *BMC Genomics* 18:640. 10.1186/s12864-017-3968-z 28830340 PMC5568309

[B49] Mehrkanoon S. Boonstra T. W. Breakspear M. Hinder M. Summers J. J. (2016). Upregulation of cortico-cerebellar functional connectivity after motor learning. *Neuroimage* 128 252–263. 10.1016/j.neuroimage.2015.12.052 26767943

[B50] Miall R. C. Robertson E. M. (2006). Functional imaging: is the resting brain resting? *Curr. Biol.* 16 R998–R1000. 10.1016/j.cub.2006.10.041 17141608 PMC6010146

[B51] Nashiro K. Sakaki M. Braskie M. N. Mather M. (2017). Resting-state networks associated with cognitive processing show more age-related decline than those associated with emotional processing. *Neurobiol. Aging* 54 152–162. 10.1016/j.neurobiolaging.2017.03.003 28390824 PMC5799084

[B52] Nasreddine Z. S. Phillips N. A. Bedirian V. Charbonneau S. Whitehead V. Collin I. (2005). The Montreal cognitive assessment, MoCA: a brief screening tool for mild cognitive impairment. *J. Am. Geriatr. Soc.* 53 695–699. 10.1111/j.1532-5415.2005.53221.x 15817019

[B53] Nichols T. Brett M. Andersson J. Wager T. Poline J. B. (2005). Valid conjunction inference with the minimum statistic. *Neuroimage* 25 653–660. 10.1016/j.neuroimage.2004.12.005 15808966

[B54] Oldfield R. C. (1971). The assessment and analysis of handedness: the Edinburgh inventory. *Neuropsychologia* 9 97–113. 10.1016/0028-3932(71)90067-4 5146491

[B55] O’Shea J. Sebastian C. Boorman E. D. Johansen-Berg H. Rushworth M. F. (2007). Functional specificity of human premotor-motor cortical interactions during action selection. *Eur. J. Neurosci.* 26 2085–2095. 10.1111/j.1460-9568.2007.05795.x 17868374

[B56] Park D. C. Polk T. A. Park R. Minear M. Savage A. Smith M. R. (2004). Aging reduces neural specialization in ventral visual cortex. *Proc. Natl. Acad. Sci. U.S.A.* 101 13091–13095. 10.1073/pnas.0405148101 15322270 PMC516469

[B57] Pauwels L. Vancleef K. Swinnen S. P. Beets I. A. (2015). Challenge to promote change: both young and older adults benefit from contextual interference. *Front. Aging Neurosci.* 7:157. 10.3389/fnagi.2015.00157 26321950 PMC4531253

[B58] Peltier S. J. LaConte S. M. Niyazov D. M. Liu J. Z. Sahgal V. Yue G. H. (2005). Reductions in interhemispheric motor cortex functional connectivity after muscle fatigue. *Brain Res.* 1057 10–16. 10.1016/j.brainres.2005.06.078 16140287

[B59] Power J. D. Mitra A. Laumann T. O. Snyder A. Z. Schlaggar B. L. Petersen S. E. (2014). Methods to detect, characterize, and remove motion artifact in resting state fMRI. *Neuroimage* 84 320–341. 10.1016/j.neuroimage.2013.08.048 23994314 PMC3849338

[B60] Pravata E. Sestieri C. Mantini D. Briganti C. Colicchio G. Marra C. (2011). Functional connectivity MR imaging of the language network in patients with drug-resistant epilepsy. *AJNR Am. J. Neuroradiol.* 32 532–540. 10.3174/ajnr.A2311 21163879 PMC8013113

[B61] Raichle M. E. MacLeod A. M. Snyder A. Z. Powers W. J. Gusnard D. A. Shulman G. L. (2001). A default mode of brain function. *Proc. Natl. Acad. Sci. U.S.A.* 98 676–682. 10.1073/pnas.98.2.676 11209064 PMC14647

[B62] Rouiller E. M. Babalian A. Kazennikov O. Moret V. Yu X. H. Wiesendanger M. (1994). Transcallosal connections of the distal forelimb representations of the primary and supplementary motor cortical areas in macaque monkeys. *Exp. Brain Res.* 102 227–243. 10.1007/BF00227511 7705502

[B63] Ruddy K. L. Leemans A. Carson R. G. (2017). Transcallosal connectivity of the human cortical motor network. *Brain Struct. Funct.* 222 1243–1252. 10.1007/s00429-016-1274-1 27469272 PMC5368198

[B64] Salvador R. Suckling J. Coleman M. R. Pickard J. D. Menon D. Bullmore E. (2005). Neurophysiological architecture of functional magnetic resonance images of human brain. *Cereb. Cortex* 15 1332–1342. 10.1093/cercor/bhi016 15635061

[B65] Sami S. Robertson E. M. Miall R. C. (2014). The time course of task-specific memory consolidation effects in resting state networks. *J. Neurosci.* 34 3982–3992. 10.1523/JNEUROSCI.4341-13.2014 24623776 PMC3951697

[B66] Sampaio-Baptista C. Filippini N. Stagg C. J. Near J. Scholz J. Johansen-Berg H. (2015). Changes in functional connectivity and GABA levels with long-term motor learning. *Neuroimage* 106 15–20. 10.1016/j.neuroimage.2014.11.032 25463472 PMC4405007

[B67] Santos Monteiro T. Beets I. A. M. Boisgontier M. P. Gooijers J. Pauwels L. Chalavi S. (2017). Relative cortico-subcortical shift in brain activity but preserved training-induced neural modulation in older adults during bimanual motor learning. *Neurobiol. Aging* 58 54–67. 10.1016/j.neurobiolaging.2017.06.004 28708977

[B68] Seidler R. D. Bernard J. A. Burutolu T. B. Fling B. W. Gordon M. T. Gwin J. T. (2010). Motor control and aging: links to age-related brain structural, functional, and biochemical effects. *Neurosci. Biobehav. Rev.* 34 721–733. 10.1016/j.neubiorev.2009.10.005 19850077 PMC2838968

[B69] Serbruyns L. Gooijers J. Caeyenberghs K. Meesen R. L. Cuypers K. Sisti H. M. (2015). Bimanual motor deficits in older adults predicted by diffusion tensor imaging metrics of corpus callosum subregions. *Brain Struct. Funct.* 220 273–290. 10.1007/s00429-013-0654-z 24158531

[B70] Serrien D. J. (2008). Coordination constraints during bimanual versus unimanual performance conditions. *Neuropsychologia* 46 419–425. 10.1016/j.neuropsychologia.2007.08.011 17904169

[B71] Serrien D. J. (2009). Functional connectivity patterns during motor behaviour: the impact of past on present activity. *Hum. Brain Mapp.* 30 523–531. 10.1002/hbm.20518 18095281 PMC6870694

[B72] Serrien D. J. Swinnen S. P. Stelmach G. E. (2000). Age-related deterioration of coordinated interlimb behavior. *J. Gerontol. B Psychol. Sci. Soc. Sci.* 55 295–303. 10.1093/geronb/55.5.P295 10985294

[B73] Shumway R. H. Stoffer D. S. (2006). *Time Series Analysis and Its Applications.* New York, NY: Springer 520.

[B74] Sisti H. M. Geurts M. Clerckx R. Gooijers J. Coxon J. P. Heitger M. H. (2011). Testing multiple coordination constraints with a novel bimanual visuomotor task. *PLOS ONE* 6:e23619. 10.1371/journal.pone.0023619 21858185 PMC3157395

[B75] Sisti H. M. Geurts M. Gooijers J. Heitger M. H. Caeyenberghs K. Beets I. A. (2012). Microstructural organization of corpus callosum projections to prefrontal cortex predicts bimanual motor learning. *Learn. Mem.* 19 351–357. 10.1101/lm.026534.112 22837217

[B76] Solesio-Jofre E. Serbruyns L. Woolley D. G. Mantini D. Beets I. A. Swinnen S. P. (2014). Aging effects on the resting state motor network and interlimb coordination. *Hum. Brain Mapp.* 35 3945–3961. 10.1002/hbm.22450 24453170 PMC6869293

[B77] Stevens W. D. Buckner R. L. Schacter D. L. (2010). Correlated low-frequency BOLD fluctuations in the resting human brain are modulated by recent experience in category-preferential visual regions. *Cereb. Cortex* 20 1997–2006. 10.1093/cercor/bhp270 20026486 PMC2901023

[B78] Swinnen S. P. (1998). Age-related deficits in motor learning and differences in feedback processing during the production of a bimanual coordination pattern. *Cogn. Neuropsychol.* 15 439–466. 10.1080/026432998381104 28657466

[B79] Swinnen S. P. (2002). Intermanual coordination: from behavioural principles to neural-network interactions. *Nat. Rev. Neurosci.* 3 348–359. 10.1038/nrn807 11988774

[B80] Swinnen S. P. Van Langendonk L. Verschueren S. Peeters G. Dom R. De Weerdt W. (1997). Interlimb coordination deficits in patients with Parkinson’s disease during the production of two-joint oscillations in the sagittal plane. *Mov. Disord.* 12 958–968. 10.1002/mds.870120619 9399221

[B81] Talelli P. Waddingham W. Ewas A. Rothwell J. C. Ward N. S. (2008). The effect of age on task-related modulation of interhemispheric balance. *Exp. Brain Res.* 186 59–66. 10.1007/s00221-007-1205-8 18040671 PMC2257995

[B82] Tambini A. Ketz N. Davachi L. (2010). Enhanced brain correlations during rest are related to memory for recent experiences. *Neuron* 65 280–290. 10.1016/j.neuron.2010.01.001 20152133 PMC3287976

[B83] Taubert M. Lohmann G. Margulies D. S. Villringer A. Ragert P. (2011). Long-term effects of motor training on resting-state networks and underlying brain structure. *Neuroimage* 57 1492–1498. 10.1016/j.neuroimage.2011.05.078 21672633

[B84] Todd G. Kimber T. E. Ridding M. C. Semmler J. G. (2010). Reduced motor cortex plasticity following inhibitory rTMS in older adults. *Clin. Neurophysiol.* 121 441–447. 10.1016/j.clinph.2009.11.089 20071228

[B85] Tung K. C. Uh J. Mao D. Xu F. Xiao G. Lu H. (2013). Alterations in resting functional connectivity due to recent motor task. *Neuroimage* 78 316–324. 10.1016/j.neuroimage.2013.04.006 23583747 PMC3672369

[B86] Ungerleider L. G. Doyon J. Karni A. (2002). Imaging brain plasticity during motor skill learning. *Neurobiol. Learn. Mem.* 78 553–564. 12559834 10.1006/nlme.2002.4091

[B87] Vahdat S. Darainy M. Milner T. E. Ostry D. J. (2011). Functionally specific changes in resting-state sensorimotor networks after motor learning. *J. Neurosci.* 31 16907–16915. 10.1523/JNEUROSCI.2737-11.2011 22114261 PMC3260885

[B88] van den Heuvel M. P. Hulshoff Pol H. E. (2010). Exploring the brain network: a review on resting-state fMRI functional connectivity. *Eur. Neuropsychopharmacol.* 20 519–534. 10.1016/j.euroneuro.2010.03.008 20471808

[B89] van Dijk V. F. Delnoy P. Smit J. J. J. Ramdat Misier R. A. Elvan A. van Es H. W. (2017). Preliminary findings on the safety of 1.5 and 3 Tesla magnetic resonance imaging in cardiac pacemaker patients. *J. Cardiovasc. Electrophysiol.* 28 806–810. 10.1111/jce.13231 28429537

[B90] Voelcker-Rehage C. Alberts J. L. (2007). Effect of motor practice on dual-task performance in older adults. *J. Gerontol. B Psychol. Sci. Soc. Sci.* 62 141–148. 10.1093/geronb/62.3.p141 17507581

[B91] Voelcker-Rehage C. Willimczik K. (2006). Motor plasticity in a juggling task in older adults-a developmental study. *Age Ageing* 35 422–427. 10.1093/ageing/afl025 16690635

[B92] Voss M. W. Erickson K. I. Chaddock L. Prakash R. S. Colcombe S. J. Morris K. S. (2008). Dedifferentiation in the visual cortex: an fMRI investigation of individual differences in older adults. *Brain Res.* 1244 121–131. 10.1016/j.brainres.2008.09.051 18848823

[B93] Waites A. B. Stanislavsky A. Abbott D. F. Jackson G. D. (2005). Effect of prior cognitive state on resting state networks measured with functional connectivity. *Hum. Brain Mapp.* 24 59–68. 10.1002/hbm.20069 15382248 PMC6871664

[B94] Wilson J. K. Baran B. Pace-Schott E. F. Ivry R. B. Spencer R. M. (2012). Sleep modulates word-pair learning but not motor sequence learning in healthy older adults. *Neurobiol. Aging* 33 991–1000. 10.1016/j.neurobiolaging.2011.06.029 22244090 PMC3307877

[B95] Wishart L. R. Lee T. D. Cunningham S. J. Murdoch J. E. (2002). Age-related differences and the role of augmented visual feedback in learning a bimanual coordination pattern. *Acta Psychol.* 110 247–263. 12102108 10.1016/s0001-6918(02)00036-7

[B96] Woolley D. G. Mantini D. Coxon J. P. D’Hooge R. Swinnen S. P. Wenderoth N. (2015). Virtual water maze learning in human increases functional connectivity between posterior hippocampus and dorsal caudate. *Hum. Brain Mapp.* 36 1265–1277. 10.1002/hbm.22700 25418860 PMC6869087

[B97] Wu J. Srinivasan R. Kaur A. Cramer S. C. (2014). Resting-state cortical connectivity predicts motor skill acquisition. *Neuroimage* 91 84–90. 10.1016/j.neuroimage.2014.01.026 24473097 PMC3965590

[B98] Yoo K. Sohn W. S. Jeong Y. (2013). Tool-use practice induces changes in intrinsic functional connectivity of parietal areas. *Front. Hum. Neurosci.* 7:49. 10.3389/fnhum.2013.00049 23550165 PMC3582314

[B99] Zar J. H. (1998). *Biostatistical Analysis.* New York, NY: Prentice-Hall 450.

[B100] Zhang H. Long Z. Ge R. Xu L. Jin Z. Yao L. (2014). Motor imagery learning modulates functional connectivity of multiple brain systems in resting state. *PLOS ONE* 9:e85489. 10.1371/journal.pone.0085489 24465577 PMC3894973

[B101] Zhang H. Zhang Y. J. Duan L. Ma S. Y. Lu C. M. Zhu C. Z. (2011). Is resting-state functional connectivity revealed by functional near-infrared spectroscopy test-retest reliable? *J. Biomed. Opt.* 16:067008. 10.1117/1.3591020 21721829

[B102] Zuo X. N. Kelly C. Di Martino A. Mennes M. Margulies D. S. Bangaru S. (2010). Growing together and growing apart: regional and sex differences in the lifespan developmental trajectories of functional homotopy. *J. Neurosci.* 30 15034–15043. 10.1523/JNEUROSCI.2612-10.2010 21068309 PMC2997358

